# Unmasking the Ethical Dimensions of Data-sharing in Health Research: Perspectives from Researchers at a Public University in Uganda

**DOI:** 10.21203/rs.3.rs-5204585/v1

**Published:** 2024-10-28

**Authors:** Paul Kutyabami, Herbert Muyinda, Moses Mukuru, Erisa Mwaka, Kamba Pakoyo, Joan Kalyango, Nelson K. Sewankambo

**Affiliations:** Makerere University; Makerere University; Nottingham University Trent; Makerere University; Busitema University; Makerere University; Makerere University

**Keywords:** data-sharing, ethical concerns, health research, Researchers, Autonomy, incentives, control, power asymmetry, Uganda

## Abstract

**Background:**

In resource-limited settings like Uganda, ethical sharing of health research data is crucial for advancing scientific knowledge. Despite the growing trend of data sharing in the digital age, its adoption in low-resource contexts is often hampered by complex ethical considerations. This report investigates these ethical concerns using data from researchers at a public university, with the goal of informing the development of practical solutions to promote ethical data-sharing practices in Uganda

**Methods:**

A qualitative phenomenographic study was conducted with 26 participants at Makerere University College of Health Sciences, including professors, lecturers, research fellows, and PhD students. In-depth interviews were conducted via Zoom or in person, using an interview guide. Data were analyzed thematically using ATLAS.ti (V9), following both deductive and inductive approaches

**Results:**

The study revealed a complex landscape of data-sharing practices among researchers. Participants had varying understandings of data sharing, with some expressing limited awareness. Incentives were widely recognized as crucial for encouraging data sharing. While acknowledging data sources in publications was appreciated, some researchers advocated for co-authorship for significant contributions. Researchers’ autonomy and control over data-sharing practices were influenced by factors such as research concept origination, funding sources, researchers’ financial status, and analytical skills. Institutional policies, cultural norms, and customs that promote a ‘siloed’ research environment also significantly influenced of data-sharing behavior

**Conclusion:**

This study revealed a complex landscape of data-sharing practices among researchers. The varying interpretations of data sharing highlight the need for enhanced education and awareness regarding its importance. The identified incentives, such as financial rewards and co-authorship, which encourage data sharing, suggest a need to establish a fair data-sharing reward system. Additionally, policies that facilitate researchers’ autonomy and data control, while fostering trust, are crucial to address the siloed research culture.

## Introduction

Ethical data sharing is essential for advancing health research, especially in low- and middle-income countries (LMICs) [1, 2]. It involves granting researchers or organizations outside the ownership circle access to datasets for further research or other approved purposes [3]. The digital age has amplified the value of data by making it reusable and shareable, thus enhancing groundbreaking discoveries [4]. By granting researchers access to datasets, data sharing enables the exploration of new research questions and facilitates timely solutions. The increasing importance of data in research has transformed data sharing from a desirable practice to a fundamental expectation within the scientific community [5, 6]. Despite this, many researchers fail to recognize the potential value of their data for future research and often neglect to preserve or share it after studies conclude [6, 7]. Given the limited research investment in sub-Saharan Africa (SSA), data sharing presents a valuable opportunity to boost research productivity. To maximize its benefits, it’s crucial to streamline data-sharing ethics. The growing need for researchers to share data beyond collaborators, including those who don’t own or directly participate in the generating project, raises ethical concerns. The moral integrity with which shared data is managed and used significantly influences researchers’ willingness to share their data [8].

The iconic phrase, “data is the new oil” underscores the significance of data across various fields, including health research [4]. Michael Palmer extended this analogy, comparing research data to crude oil: both have inherent value that can only be realized through refinement. [9]. Just as crude oil needs to be transformed into valuable products like gas, plastic, and chemicals, data must be analyzed to extract valuable insights [9]. The diversity of analytical processes and interpretations applied to research data determines the breadth and impact of the resulting insights, maximizing its utility [10–14]. One key factor enabling optimal data exploitation is sharing. Unlike oil, a finite resource, data is non-rival and durable [15, 16]. This means multiple users can access and reuse it simultaneously without interfering with each other’s activities and it can be processed repeatedly without diminishing its value [16, 17]. These characteristics make data amenable to and ideal for sharing.

However, the recognition of the value of data and data-sharing have not been uniformly diffused globally. There remains untapped potential, particularly in LMICs like Uganda [18–20].

Research in SSA continues to be underfunded compared to the rest of the world. Africa accounts for 1% of global research investment and contributes only 1.1% to global research data [21]. There are notable disparities across countries in SSA, for example, Nigeria, Egypt, and South Africa make up 65.7% of Africa’s research investment [21, 22]. To increase research investment in Africa, the African Union recommended the allocation of at least 1% of GDP to research and development, but no African nation has achieved this target. On average, Africa invests less in research (0.42% of GDP) compared to the global average (1.7%) [23, 24]. Consequently, it’s crucial to find alternative strategies to enhance research output amidst the low investment.

Despite limited research investment in SSA, data remains underutilized due to low data-sharing adoption. This paper presents researchers’ experiences and the ethical concerns about data sharing in this low-middle-income setting.

## Methods

### Study Design and Site

This study used a phenomenographic approach to investigate health researchers’ perspectives on the ethical concerns of data sharing at the Makerere University College of Health Sciences (MakCHS). Unlike phenomenology, which focuses on lived experiences, phenomenography allows us to examine how individuals understand and perceive a phenomenon, even if they haven’t personally experienced it [25, 26]. This approach was chosen to capture the diversity of perceptions on data sharing, even among researchers without personal experience. By understanding researchers’ ethical concerns, regardless of their experience, we aimed to gain insights into the broader issue of data sharing in the health research context.

The study was conducted at MakCHS, renowned for its contribution to health research in Uganda and the region. According to the 2019 Makerere University Annual Report, MakCHS contributes 50% of the university’s research output and 70% of the nation’s health research publications in Uganda [27]. Makerere University stands as a prominent African research institution, and contributes 1.8% of Africa’s research publications [28].

### Sampling and Recruitment

Twenty-six participants were purposefully selected based on their research experience, discipline, school affiliation, and position. These included professors, lecturers, research fellows, and PhD students.

The principal investigator (PK) contacted Deans and Heads of Departments to obtain participant lists. Potential participants were then contacted by phone or email to schedule Zoom or in-person interviews.

### Demographics of Participants

The demographic profile of participants in this study reflects a diverse range of backgrounds and positions within the academic and research community (Table 1). Most were male, over 50 years, and held senior lecturer positions or higher. The majority were from the School of Medicine and held PhDs. All participants reported receiving external public funding, with some also receiving private funding. A few used personal savings for their research. Data was mostly stored on paper and when digitized, on laptops with passwords and in some cases electronic servers. See Table 1.

#### Tools and Data Collection

A comprehensive interview guide was developed to explore participants’ educational backgrounds, research involvement, and data-sharing practices. It included open-ended questions and probes to facilitate in-depth discussions. The guide was pilot-tested with four healthcare professionals at Uganda’s Mulago National Referral and Teaching Hospital who were not staff of the MakCHS.

PK conducted the in-depth interviews in English, either via Zoom or in-person. Informed consent was obtained electronically for Zoom interviews and in paper form for in-person sessions. Interviews lasted 45–74 minutes and were recorded with participants’ permission.

### Data Analysis

The data analysis process began with a meticulous transcription of all interview recordings into Word documents by PK. To ensure accuracy and reliability, another person independently reviewed the transcripts while listening to the audio recordings. A hybrid thematic analysis was conducted, combining deductive and inductive approaches[29, 30]. PK, led the analysis, with qualitative data analysis expertise input from MM and HM.

**Figure 1:**
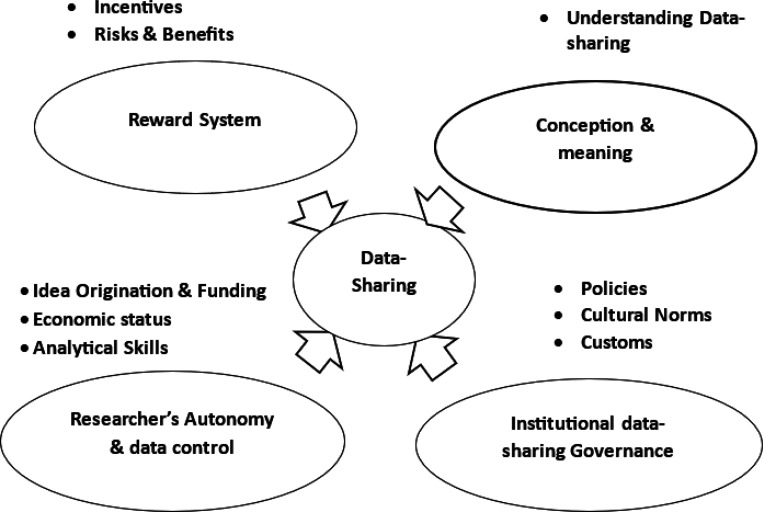
Visual representation of themes and subthemes

First, PK immersed himself in the data by reviewing the transcripts multiple times, drawing on his firsthand experience from the interviews and transcription. This deep engagement allowed him to gain a nuanced understanding of the participants’ perspectives and experiences. In the second step, PK collaborated with qualitative data analysis experts, MM and HM, to start the coding process. Using three randomly selected transcripts, the team employed a hybrid coding strategy, combining a priori codes from the literature review (deductive) with open coding to identify emergent themes (inductive). This approach ensured a comprehensive and systematic analysis, capturing both predetermined and unexpected themes. Subsequently, in the third step, through collaborative discussions and consensus building, the team established a finalized codebook encompassing a comprehensive set of codes. This codebook was then used to systematically code all transcripts using ATLAS.ti (v9). Codes were assigned to relevant sections of the text, capturing the key ideas and concepts expressed by the participants. In step four, codes were organized into subthemes and higher-order themes. This involved grouping related codes and identifying overarching patterns or concepts that emerged from the data. See Fig. 1.

In the fifth step, the identified themes were refined and further developed through a process of analysis and interpretation. This involved examining the relationships between themes, identifying variations within themes, and exploring the nuances of the data. The final step involved interpreting the identified themes and developing a narrative description for each theme. All themes and their descriptions were then collated to provide an overall description of the results.

### Ethical considerations

#### Ethical approval

was obtained from the School of Medicine Research and Ethics Committee (Ref no. Mak-SOMREC-2021–89) and the Uganda National Council for Science and Technology (Ref no. HS263ES). Prior to participation, written informed consent was obtained from each participant. To ensure anonymity and confidentiality, participant codes were used throughout the study.

## Results

Four key themes emerged: conception and meaning of data sharing, reward system for data-sharing, researchers’ autonomy and data control, and institutional data sharing governance

### 1. Conception and meaning of data-sharing

The understanding, meaning and implications of data-sharing varied among the participants. While most researchers demonstrated a comprehensive grasp of the concept, some expressed limited clarity. A professor holding an administrative position, IDIR01 defined data-sharing as, “*making data accessible to other people who could potentially use that data*.” Data-sharing’ was thus understood as both a means and outcome of making data accessible to other people who could potentially use it. It was seen as a process involving the transfer of ownership, ceding of power, loss of control, and expectations associated with allowing others (often unofficial) to use the data. At the same time, other participants illustrated limited knowledge, for instance one mentioned, *“I must admit, I’m not very familiar with it, and I’m not entirely confident in my understanding.”* (IDIR12, a PhD student)

Some researchers viewed data sharing as a means to foster research collaborations. They observed that making datasets available for sharing draws potential collaborators and promotes collaboration. As Professor IDIR20, with large portfolio of collaborations, expressed *“The more you open up your data for others to use, the more you can attract collaborators to work with you.”* Others however considered the intersection between collaboration, data sharing, and data ownership. They questioned whether using the same dataset within a collaborative project constituted data-sharing or data co-ownership. A professor argued that data-sharing involves providing data to external parties, not collaborators, stating,

“...if you are involved in a collaboration, and your collaborators are utilizing the data you generated together, I believe that could be considered co-ownership. However, in the strictest sense, data-sharing involves providing my data to someone anonymous” (IDIR22).

### 2. Unfair Reward System for Data-Sharing

#### Incentives

Researchers highlighted the general unfairness of the current reward system, noting that data-sharing thrives when supported by appropriate incentives. They stressed the importance of mechanisms to reward those who invest time and resources in creating valuable datasets. While formal acknowledgment in publications was seen as a positive step, many researchers believe that co-authorship on publications that utilize their data is a more equitable and meaningful incentive.

As a PhD fellow remarked, *“If they acknowledged my contribution, I wouldn’t have any problem with it. Because that is what research is all about”* (IDIR02). *On the same note, a junior researcher suggested, “People who access secondary data need to take an extra step, not merely acknowledgement but reaching out to those who share data and exploring the possibility of co-authorship…”* (IDIR13).

Participants also observed that the current research assessment system, heavily focuses on publications and grants, and often overlooks data-sharing contributions. Participants proposed incorporating data-sharing metrics into research evaluations. As IDIR13 a junior researcher noted,

“…if we implement a scoring system that values data-sharing leading to publications, it not only elevates the institution’s profile but also warrants recognition and rewards for individuals within the promotion criteria…”

The concept of a tiered reward system based on researchers’ financial investment in data generation was also suggested but this provoked debate. Some especially those who invested personal funds believed that such a system would incentivize data sharing. As a recently graduated PhD student stated,

“Data collection often involves significant costs, and I incurred some of those out-of-pocket expenses for my research. I believe researchers who contribute to data collection should be compensated. Perhaps a percentage of funding could be allocated for data collection contributions, or a separate fund to support those who invest their resources in research.” (IDIR12)

However, others like IDIR20 a junior staff experienced in research regulation, argued that the inherent benefits of research, such as career advancement and professional development, already compensate self-funded researchers. He noted that, Even if researchers invest personal funds, they have gained valuable experience by completing their research, answering their research question, and potentially publishing the findings. This, in itself, serves as a form of compensation.

#### Risks and Benefits

Researchers in this study opined that incentives alone might not be sufficient to encourage data sharing. They emphasized the importance of carefully considering risks and benefits before sharing data, even with incentives in place. Several benefit and risks data-sharing were highlighted including; increased efficiency in resources utilization, fosters collaboration, and bolstering research integrity.

Researchers mentioned that sharing data eliminates the unnecessary duplication of research activities thus saving resources and reducing the burden to both researchers and participants, as a very experienced researcher stated, *“Using existing datasets can save a lot of time and finances as well as being safe to participants, and less cumbersome to researchers.”* (IDIR06)

It also enhances data utility and increases research output. As a junior researcher IDIR13 aptly stated, “*We can utilize the same data set to investigate many questions.”* Failing to share data can lead to a waste of resources, as further noted by IDIR13, *“...sometimes you hold on to data thinking, oh, I’ll use it, and then you actually don’t really use it. And you know, it’s wasted resources.”*

Additionally, sharing data enables researchers to gain a more comprehensive understanding of research problems from diverse perspectives, facilitating quicker resolution of societal issues. As a researcher noted, *“Data-sharing enables us to tackle research problems from multiple angles, enriching our understanding and ultimately contributing to addressing societal challenges.”* (IDIR13)

Researchers also considered data-sharing as a form of peer review that promotes data integrity, as IDIR02, a PhD student articulated: *“Data sharing allows scrutiny of data quality and the interaction between researchers can further enhance data integrity.”*

While data sharing offers significant benefits, researchers also recognize potential risks. These include data misuse, privacy breaches, loss of intellectual property, exploitation, unwarranted scrutiny, unauthorized use, insufficient attribution, and loss of control over their data. Balancing these risks against the benefits is crucial when deciding to share data.

Researchers expressed concern that data sharing could invite undue scrutiny, potentially damaging their reputation. As IDIR02 noted, *“Someone accessing data with the intent to find flaws could lead to criticism of the research and cast doubt on findings, harming the researcher’s reputation.”*

Data sharing also reduces one’s control over data access, raising concerns about unauthorized use. As IDIR12, a recent PhD graduate narrated her experience, *“I shared data with a colleague, who then shared it with others without my permission.”*

Researchers also expressed concerns over the possibility of exploitation, where individuals might access data and use it without proper attribution or equitable sharing of benefits. As a researcher IDIR21 shared an experience, *“People fear data theft. Accessible data can be used to write a paper before you do. I presented data at a conference and later saw a published paper with my data but without my name.”*

### 3. Researchers’ Autonomy and Data Control

The study examined the factors that influence researchers’ involvement in making data-sharing decisions. Findings revealed that researchers’ autonomy is often constrained by various factors, such as the source of the research idea, funding mechanisms, economic status, and analytical capabilities. These factors can affect data ownership, access control, and subsequent reuse.

#### Research Funding Mechanism and Research Ideas Origination

Researchers reported that their autonomy in data-sharing decisions is often influenced by external factors, especially where external funding or research concept are involved. Funding agencies and individuals who conceive research ideas frequently shape data-sharing practices. The analysis revealed that

When researchers independently conceive and pursue their research projects, they often seek external funding to support their work. Such funding often comes with conditions, Including data ownership and access requirements. As Professor involved in international collaborative research participant remarked

“Data-sharing has become a prerequisite for both obtaining funding and publishing research. “ (IDIR20).

However, the extent to which funders influence researchers’ data-sharing decisions varied significantly. Some funders grant researchers ownership and data control rights while others don’t. For example, a PhD student recounted, *“After we collected the data and had the dataset, someone expressed interest in using it in their publication. However, we declined, claiming that the data could not be released until our research was published.”* (IDIR02)

When funders initiate the research idea, conceptualize the research and contract researchers to carry it out, data ownership typically resides with the funder. As one professor in the late 60s (IDIR18) explained, *“ Right from the start, they make it clear that the data belongs to the funder. They allow us to contribute to the writing, but the data remains under the funder’s control.”*

Conversely, when researchers conceive the research idea and implement the project using personal savings, they generally have greater control over data sharing decisions.

#### Economic Status

This study found that researchers’ economic status significantly influenced their ability to share data effectively. Those with limited funding often resorted to less secure methods, such as storing data on personal laptops or in locked cabinets. This not only hindered long-term preservation but also made data difficult to share and access. As IDIR13, a researcher having self-funded projects explained,

“I have a dedicated cabinet funded by the project for data storage, and I also keep some qualitative data in electronic format on my computer.”

While these methods were feasible for individual researchers, they present significant barriers to long-term data preservation and shareability, requiring researchers’ physical presence to access data. Paper records were particularly vulnerable to threats like theft, fire, and even abandonment. IDIR13 further recounted a tragic experience: *“We had data on paper that a colleague had kept, but when I needed it, some of it had already been destroyed.”* In another incident, the same researcher’s laptop crashed, preventing him from sharing data with people who requested it after reading his published paper.

Conversely, well-funded researchers often stored data electronically on servers. This approach offered numerous advantages, including long-term preservation, easy access and facilitated collaboration among research partners. A professor involved in several collaborations, IDIR06 emphasized the importance of this method, stating,

We store electronic data on servers… We ensure both our partners and ourselves have copies.

Therefore, when funding agencies provided better data storage technologies, they gained more influence over data-sharing decisions. Such technology allows funders to dictate the terms and conditions for data sharing while limiting researcher input.

#### Analytical Capabilities

The disparity in analytical skills and access to advanced data analysis tools created an uneven playing field in research, favoring researchers with better capabilities and resources. In data-sharing collaborations, researchers with superior analytical capacities were often able to process data more quickly and efficiently, giving them a significant advantage over those with limited funding. As one researcher (IDIR21) expressed: *“Sharing data is risky. Someone could easily write a paper with your data before you. Raw data offers no ownership claim. It’s happened to me. We discussed data, and then a paper emerged from my data without my name.”*

This disparity often made researchers with limited resources hesitant to collaborate and share data fearing exploitation.

### 4. Institutional Data Sharing Governance

Institutional data governance, comprising of policies, cultural norms, and customs, significantly influenced the ethical landscape of data sharing. These factors define roles, responsibilities, and decision-making processes related to data-sharing. While policies provide formal guidelines, cultural norms and customs shape the informal expectations and behaviours that impact data-sharing practices.

Together, they significantly influence researchers’ willingness and approaches to data sharing within institutions, often prioritizing sole ownership of research data and discouraging data-sharing.

#### Institutional culture

Researchers reported that the prevailing cultural norms view research as a linear process involving idea conception, data collection, and exclusive researcher use of the data. This mindset is often instilled during academic training, as one influential professor noted,

“Since our undergraduate years, we’ve believed that researchers conceive their work, gather data, and use it exclusively. The notion of sharing was absent from this equation. The culture at our university, including the MakCHS, has long embraced this perspective.” (IDIR06)

This stereotype within some academic circles can stigmatize researchers who leverage existing data, portraying them as lazy or lacking essential research skills. As an experienced researcher pointed out:

“When someone relies solely on existing data, questions arise about whether they acquire comprehensive research skills, extending beyond data analysis. This encompasses ethical research conduct and community involvement. Sharing data opens the door for anyone, including researchers perceived as lazy.” (IDIR06)

#### Sociality in data-sharing

Researchers reported that the quality of social relations influence data-sharing. It was easier for the researchers to shared data with persons they had good quality relationships with, such as trusted colleagues, students they supervise, or close friends. However, this trend, restricted data sharing to confined tightly knitted groups, creating “silos” of information. A recent PhD graduate highlighted this issue, stating, *“The way research is conducted currently... seems to be in silos”* (IDIR14). This sentiment was corroborated by others, notably those who shared data with their students. One professor, emphasized that for every research project, he collaborates with at least one PhD candidate and three master’s degree students by providing them with data (IDIR06).

Participants described this as a discriminatory and isolating culture, limiting access to key datasets for researchers outside these social networks. As IDIR14 a middle level researcher eloquently put it, *“You cannot share what you don’t know whether it exists or it doesn’t exist.”*

Even on rare occasions when researchers attempted to access data, they encountered resistance and significant delays. One professor recounted a frustrating experience:

“I sought access to a colleague’s dataset after he published two papers. Unfortunately, permission to share it was granted three years later, rendering my intended analysis impossible.” (IDIR13)

#### Institutional policies and Guidelines

Researchers reported a lack of clear national and institutional guidelines or policies governing data sharing. This created a chaotic and unpredictable environment for researchers, hindering collaboration and potentially compromising the ethical data-sharing. This fostered the perception that data sharing is an optional, altruistic act rather than an ethical responsibility. As middle career researcher stated,

“There’s no clear policy to share data, and many institutions don’t support it. This creates an impression that data sharing is a benevolent act and not an ethical obligation.” (IDIR14)

Without a standardized framework, researchers faced challenges in negotiating data-sharing agreements with partners, even when involving legal departments. Researchers felt vulnerable to exploitation and susceptible to the demands of funders and collaborators. As explained by a professor in an administrative position,

“By solely relying on external funders, we become overly vulnerable. Without clear national and institutional policies, researchers lack the necessary safeguards. This dependence on external actors hinders our ability to protect our interests and resources.” (IDIR19)

The absence of clear policies has contributed to recent disputes over research outcomes and benefits, highlighting the urgent need for national and institutional guidelines to govern data-sharing practices.

“Given the increased investment of public funds in research, clear guidelines are imperative. Recent instances of substantial research funding with limited public benefit underscore the urgent need for clear policies and stakeholder engagement.” (IDIR19)

## Discussion

The study examined ethical considerations surrounding data-sharing at MakCHS through four themes: the meaning of data sharing, rewards for sharing data, researcher autonomy and control of data, and institutional data-sharing governance. These themes were analyzed in relation to the ethical principles of respect for autonomy and justice. Additionally, the study explored how the coloniality of knowledge contributes to epistemic injustice, perpetuating power imbalances and limiting researcher autonomy and benefit-sharing in data-sharing.

### Demographic Characteristics

The ethical implications of data-sharing in academic research are complex and influenced by factors such as researcher demographics. Our study found that older male researchers were predominant, consistent with existing literature on gender and age disparities in academic institutions, especially at senior positions [31, 32]. This demographic profile may influence perspectives on data-sharing and associated ethical concerns. Older researchers with their longer experience, were more hesitant to share data than younger ones. Similar findings were reported in a study among psychology researchers, which revealed that researchers younger than 45 years were more likely to share data than those older [33].

Hesitancy among older researchers could be attributed to unpleasant experiences that led to concerns about data misuse or exploitation, while the urge to share among younger researchers could be driven by a need to foster new collaborations [34, 35]. While Lin and wang, 2020, acknowledge gender as a factor influencing data-sharing [36], the male-dominated nature of research teams observed in this study reflects a broader issue in academia: the lack of gender diversity and equity in research leadership [37, 38]. This imbalance may influence how research resources including data are shared and utilized. Achieving more gender-balanced research teams could offer diverse encourage perspectives on data sharing and broader collaborations.

### Varying Conceptions and Meanings of Data-Sharing

In this study, researchers’ understanding of data-sharing varied widely. Some had a narrow view, limiting sharing to their collaborative networks, while others had a broader perspective, that extended sharing beyond these networks. These differing views significantly influenced their data-sharing decisions. A narrow view hinders wider data dissemination and limits generation of new perspectives on problems, whereas a broader view fosters novel insights but may raise concerns about mistrust and exploitation. Researchers with a narrow perspective or lack clarity benefits of data-sharing, may be hesitant to share data especially with unfamiliar or untrusted individuals [39]. The variation in awareness aligns with findings from studies previous studies conducted among researchers at USA Universities and Federal agencies, as well as researchers in SSA [40, 41].

Researchers’ experiences play a crucial role in shaping their understanding of data-sharing. Senior researchers with more exposure to data sharing, are likely to have a deeper understanding than early-career researcher. Their influence can be significant in shaping the behavior of early-career researchers. The influence of the researcher’s experience, is supported by a study that examined corresponding authors of cancer research articles, which found that researchers’ experiences impact their data-sharing decisions [42]. While our study suggests that experience can positively influence researchers’ intention to share data, this is likely only true if the experiences were good. Bad experiences may have the opposite effect.

### Researchers’ Autonomy and Data Control

Our findings align with Verhulst and Young’s observations that power asymmetry significantly influences data-sharing practices [43]. This imbalance is possibly driven by factors such as funding mechanisms and research idea origination, economic status, and researchers’ analytical capabilities. Some researchers expressed a sense of coercion in data-sharing relationships, often feeling pressured to share their data due to economic disadvantage relative to their funders and collaborators. This phenomenon reflects broader issues of coloniality of knowledge and epistemic injustice, which can strain research relationships in data-sharing [44–46].

The power dynamics inherent in data-sharing relationships can create a sense of unequal bargaining power, where researchers may feel obligated to comply with funders’ and or collaborators’ demands to secure funding or maintain relationships and professional standing. This can lead to situations where data is shared without adequate consideration of the researchers’ interests or concerns.

Funding sources exert varying degrees of influence on data-sharing relationships, depending on the origin of the research idea[45]. When researchers initiate a research concept and seek funding from external sources, often Western institutions, data-sharing dynamics are shaped by the funders’ policies. While these policies aim to promote research integrity and transparency, they might not perfectly align with researchers’ specific goals. Funders may have broader objectives, like advancing science or addressing societal issues, which can lead to mismatches with researchers’ more focused aims. This mismatch can create tensions, especially when funders’ policies don’t fully accommodate researchers’ needs [47–49].

One may infer than even in circumstances where funders policies don’t align with researchers’ interests, researchers still have liberty to seek alternative funders that fit their needs. However, funders using public funds might have more flexible data-sharing policies that allow researchers to control access and use of their data.

In contrast, when funders formulate the research ideas, fund the research and only contract researchers to execute the projects, funder typically retains ownership of the data collected. This often limits researchers’ autonomy in making data-sharing decisions, as the funder’s interests dictate the terms of data access and use, potentially restricting broader data sharing. This aligns with a study conducted in Ghana and Tanzania among members of the Health Demographic Surveillance System (HDSS) and Research Ethics Committees (REC) that found that data ownership claims were largely influenced by the source of funding [5]. Conversely, researchers who self-fund their work generally have greater control over their data and can exercise more autonomy regarding data sharing.

Additionally, economic disparities among researchers significantly influences power dynamics and bargaining power in data-sharing relationships. Researchers’ financial resources directly influence their ability to store, control access and reuse of data. The limited funding available in LMICs, frequently necessitates researchers to adopt less secure data storage methods, such as hard copies or personal laptops. Researchers who store data in these personal spaces, maintain more control over their data compared to those using centralized storage spaces. This observation aligns with Hall et al. (2012), who noted that data stored in centralized locations results in owners relinquishing physical control over their data [50]. While personal data storage offers researchers more autonomy, it also compromises data preservation and accessibility, making data-sharing cumbersome. Well-funded researchers, often from developed countries, can afford advanced electronic storage solutions that facilitate long-term storage and seamless data-sharing. This economic disparity not only affects the logistical aspects of data storage but also grants well-funded researchers greater ownership and control over data-sharing terms, as observed in studies among Tanzanian postgraduate students [51]. These findings support the assertion that data owners wield significant authority in granting access rights [52].

Power asymmetries in data sharing in research create a perception among LMIC researchers that the principle of justice may be undermined if LMIC researchers share data with researchers in developed countries. If data is shared, researchers from developed countries can quickly and astutely analyze and publish it before them. Instances of unauthorized data use and publication without appropriate acknowledgment exacerbate the fears of potential exploitation contributing to hesitancy in sharing data.

The dominance of Western institutions in knowledge production and economic disparity leads to epistemic injustice against researchers from LMICs [45, 53–56]. Their limited access to research tools and technologies hinders their participation and contributions to the broader research community [54, 57]. This perpetuates a cycle of injustice, disadvantaging Global South researchers by excluding or undervaluing their knowledge [45, 56, 58]. To address this, all knowledge should be valued and considered on its own merit, regardless of its origin. Excluding Southern voices prevents the creation of a truly comprehensive understanding of the world.

### Institutional Data-sharing Governance

This study identifies both formal elements (policies) and informal elements (cultures and customs) as key factors influencing data-sharing within research institutions [59]. These elements can either foster a supportive environment or creates barriers that hinder data sharing. Formal policies serve as guidelines for data ownership, access, and sharing, often outlining specific procedures for data management and dissemination. In contrast, cultural norms and customs shape informal expectations and behaviors related to data-sharing.

While policies can provide a clear framework for data sharing, their effectiveness is diminished if they do not align with established cultural norms. A supportive institutional culture fosters a climate of openness, trust, transparency, honesty, fairness, and benevolence, essential for encouraging data-sharing. Conversely, a siloed culture restricts it [60, 61]. Thus, strong cultural norms of openness provide foundation for data sharing while institutional policies offer guidance for the process. The interplay between the formal and informal elements ultimately determines the extent of data-sharing both within the institution and in the broader research community.

The absence of clear institutional policies and presence of a siloed research culture could be partly responsible for the low data-sharing practices observed in this study, underscoring the critical role that institutional cultural norms and customs play in data-sharing. A study, Barlosiu involving 34 researchers found similar trends, demonstrating how cultural norms like social relationships can impact data-sharing [62]. A research environment characterized by “siloing” confines data within closed social circles, creating opacity around data and inequity in data access, which hinders data broader utility The reluctance of researcher to share data with individuals whose motives they don’t understand shows that dishonesty erodes trust and hinders data-sharing [63, 64]. Similarly a positive association between transparency and data-sharing was identified among researchers at U.S. biopharmaceutical companies [65]. To promote fairness, justice, and collaborative research, it is essential to dismantle these silos and foster a culture of open communication and data-sharing within the research community. This will ensure that all individuals with appropriate skills have equal access to data

Additionally, the belief that research must always involve collecting new data can discourage data sharing. This misconception negatively stereotypes researchers who use existing data as lazy or lacking essential skills. As a result, this culture stigmatizes data sharing and hinders the integration of secondary data use into student research.

Misconceptions, unsupportive institutional culture norms, and a lack of explicit local policies often led researchers to share data out of obligation, motivated by external factors such as funder and journal requirements. Additionally, lead researchers may share data out of a sense of obligation. However, these external policies may not align with researchers’ personal interests, stifling their autonomy in data-sharing [66–68].

Despite institutional challenges, global trends in data-sharing and recent developments like the enactment of the Data Protection and Privacy Act (DPPA) and the establishment of Research Cloud Computing centers at Makerere University may spark interest in data-sharing and provide a foundation for developing data-sharing policies [69]. These policies should address ethical concerns, including potential risks in data-sharing, authorship, acknowledgment, and benefit-sharing, to alleviate researchers’ anxieties, and build trust in the research community[60, 70–74].

### Reward System

While researchers’ knowledge, desire for autonomy, and institutional governance of data-sharing influence their decisions, on a practical level, incentives play a crucial role in shaping their willingness to share data [75]. The influence of incentives is dualistic on researchers’ autonomy, some can support researchers’ autonomy, while others can restrict or undermine the autonomy. Supportive incentives positively reinforce autonomy by providing researchers with a range of choices. For instance, offering researchers options like data-sharing badges or financial compensation alongside robust data-sharing plans and clear control mechanisms empowers them to exercise their autonomy in sharing data [76–78]. According to our study, financial rewards and the incorporation of data-sharing into research assessment criteria serve as supporting incentives for researchers to share data. Conversely, coercive incentives, such as mandatory data-sharing requirements for funding or publication, can diminish researchers’ autonomy [79, 80]. While acknowledging that incentives promote data-sharing, Esmaeilzadeh and Mirzaei point out that these measures might not be sufficient on their own [81]. Researchers carefully weigh the benefits and risks of data-sharing before making decisions [34, 41, 72, 82, 83]. A risk-benefit analysis is essential for morally appropriate data-sharing practices [84–86].

### Strengths and Limitations

This study leverages its strengths by being conducted at a research-intensive institution, fostering collaboration and access to diverse expertise. The inclusion of staff at various levels and research backgrounds further enriches the findings by incorporating a wider range of perspectives. The phenomenographic qualitative approach allows for an in-depth exploration of both researchers with lived data-sharing experience and those without, leading to a richer understanding of the concept compared to quantitative methods.

However, limitations exist. First, excluding master’s and undergraduate students creates a gap in understanding early-career researcher perspectives. Second, we did not explore view of researchers in none academic research institutions. Third, qualitative research inherently limits the generalizability of findings to a broader population. Finally, selection bias is a possibility, as participants may hold different views than non-participants.

## Conclusion

This study explored the ethical complexities of data-sharing at MakCHS, Uganda, through four key themes: the meaning of data sharing, rewards for sharing data, researcher autonomy and control of data, and institutional data-sharing governance. These were analyzed in relation to the ethical principles of respect for autonomy and justice, revealing how coloniality of knowledge perpetuates power imbalances and epistemic injustice.

Key findings reveal a diversity of perspectives on data sharing among researchers, influenced by demographic characteristics, experience, and institutional context. Researchers expressed concerns about limited autonomy, coercion, and potential exploitation in data-sharing relationships. Power dynamics, driven by factors like funding mechanisms and economic disparities, contribute to these concerns.

Addressing these challenges requires a multifaceted approach. To promote fairness and collaboration, it is essential to dismantle siloed research culture and foster a culture of open communication and data-sharing. Institutions should establish clear data-sharing policies that align with ethical principles, promote transparency, and foster a supportive culture. Equitable reward systems, including both financial incentives and recognition, can encourage data sharing while respecting researchers’ autonomy. Finally, addressing the coloniality of knowledge and promoting epistemic justice is crucial to ensure fair and inclusive data-sharing practices.

By implementing these measures, MakCHS can create a more ethical and equitable data-sharing environment, fostering collaboration, innovation, and the advancement of research for the greater public good.

## Figures and Tables

**Table 1:** Participant demographic characteristics

Variable	Category	N
Sex	Male	20
	Female	6
Age	Above 60 years	4
	50 years – 59 years	9
	Below 50 years	13
Position	Professor	5
	Associate Professor	5
	Senior Lecturer	7
	Lecturer	5
	Assistant Lecturer	1
	PhD Student	3*
	Research Fellow	2
Highest Academic Qualification	PhD	17
	PhD Fellow	3
	Masters	6
School	Medicine	10
	Biomedical Sciences	5
	Public Health	2
	Health Sciences	6
	Dentistry	3
Funding Source^◊^	Public	26
	Private	2
	Personal Saving	2
Data Storage Method Used Most	Electronic	3
	Hybrid^⛝^	3
	Hard copy and Laptop	20

* One of the students was the Assistant Lecturer and another was a Lecturer

◊ Four of the respondents received funding from two sources

⛝ Use both electronic and hard copy data storage, but prioritize digital formats

## Data Availability

The datasets used and analyzed in this study are available upon reasonable request from the corresponding author.

## What is known about this research topic?

While it is known that data sharing in health research is generally low in low- and middle-income countries (LMICs), this information is mostly anecdotal when it comes to Uganda. Additionally, it is recognized that Uganda lacks formal guidelines and policies for data sharing.

## What does this study add to the existing literature?

This paper explores data-sharing in Uganda from the researchers’ perspective. It provides a holistic view by examining both the factors that influence data-sharing decisions and the ethical considerations that weigh on researchers’ willingness to share their data.

## Abbreviations

DPPA: Data Protection and Privacy Act
GDP: Gross Domestic Product
HDSS: Health Demographic Surveillance System
LMICs: Low- and Middle-Income Countries
MakCHS: Makerere University College of Health Sciences
SOMREC: School of Medicine Research and Ethics Committee
SSA: Sub Saharan
Africa REC: Research Ethics Committee
UNCST: Uganda National Council for Science and Technology
